# A Multi-Center Retrospective Analysis Examining the Effect of Dipeptidyl Peptidase-4 Inhibitors on Progression-Free Survival in Patients With Prostate Cancer

**DOI:** 10.7759/cureus.14712

**Published:** 2021-04-27

**Authors:** Kelsey Pan, William P Skelton, Mohammed Elzeneini, Thu-Cuc Nguyen, Aaron J Franke, Azka Ali, Rohit Bishnoi, Long Dang, Nam H Dang, Julie Kish

**Affiliations:** 1 Internal Medicine, University of Florida, Gainesville, USA; 2 Oncology, Moffitt Cancer Center, Tampa, USA; 3 Internal Medicine, Moffitt Cancer Center, Tampa, USA; 4 Hematology and Oncology, University of Florida, Gainesville, USA; 5 Oncology, Ochsner Health System, Baton Rouge, USA

**Keywords:** prostate cancer, dpp4, cd26, metformin

## Abstract

Background

Cluster of differentiation 26/dipeptidyl peptidase-4 (DPP4) is a cell surface glycoprotein with multifaceted roles, including immune regulation, glucose metabolism, and tumorigenesis. Recent literature has identified DPP4 inhibitors to improve survival in diabetic patients with prostate cancer. DPP4 inhibitors have been proposed to play a role in prostate cancer, as DPP4 is found at higher levels in malignant prostate tissue compared to benign and correlates with PSA levels and cancer stage. In this multi-center retrospective study, we aim to define the effects of DPP4 inhibitors on progression-free survival (PFS) in diabetic patients with advanced-stage prostate cancer.

Methodology

We performed a retrospective analysis of 161 patients with diabetes and advanced-stage (III or IV) prostate cancer at the University of Florida Health Cancer Center and Moffitt Cancer Center. Our cohort included 120 patients on metformin (control group) and 41 on a DPP4 inhibitor (study group).

Results

No significant difference in progression of prostate cancer was identified between those on DPP4 inhibitors versus metformin (hazard ratio [HR]: 1.01; 95% confidence interval [CI]: 0.64-1.61; p = 0.955). Median time to progression was 3.5 years (range: 2.4-4.6 years).

Conclusions

Despite prior literature indicating survival benefit of DPP4 inhibitors in prostate cancer, our study did not identify a statistically significant improvement of PFS in diabetic patients with advanced prostate cancer. Additional analysis with larger sample sizes and prospective investigation with study of tumor microenvironment are needed to evaluate clinical impact and potential survival benefit of DPP4 inhibitors in prostate cancer.

## Introduction

Dipeptidyl peptidase-4 (DPP4), also known as cluster of differentiation 26 (CD26), is a commonly expressed cell surface protein found in many cell types that can function as a tumor suppressor or activator, depending on the cancer type and its interactions with the tumor microenvironment (TME). DPP4 plays a role in chemokine signaling, immunoregulation, and glucose homeostasis. DPP4 inhibitors are most commonly utilized as a class of oral hypoglycemic drugs used in type two diabetes mellitus called gliptins. Drugs in this class include sitagliptin, saxagliptin, alogliptin, and linagliptin. Gliptins lower serum glucose levels by inhibiting DPP4, resulting in an increase in incretin levels, which thereby inhibit glucagon release and stimulate insulin release [1].

Adding to the multifaceted role of DPP4 is its role in the TME in human cancers. DPP4 has been studied as a tumor biomarker due to its expression in various primary tumors and metastases. DPP4 is expressed on numerous tumors, including malignant pleural mesothelioma (MPM), renal cell carcinoma (RCC), gastrointestinal stromal tumor (GIST), hepatocellular carcinoma (HCC), and colorectal, lung, prostate, and ovarian cancer [2]. Recent literature has established benefits of DPP4 inhibitors on progression-free survival (PFS) of diabetic patients with advanced colorectal and airway cancers, as well as improved overall survival (OS) in colorectal and lung cancer [3,4].

Shah et al. recently established that DPP4 inhibitors offer a significant survival advantage in prostate cancer [5]. Malignant prostate tissue was found to contain higher levels of DPP4/CD26 compared to benign tissue. Furthermore, DPP4 levels correlated with prostate-specific antigen (PSA) level, residual tumor, tumor size, and stage in prostate cancer. Due to these associations, DPP4/CD26 has been proposed as a potential tumor marker to predict clinical course in prostate cancer [6]. In this study, we examined the effect of DPP4 inhibitors on PFS in diabetic patients with advanced-stage prostate cancer.

## Materials and methods

We performed a multi-center retrospective analysis at the H. Lee Moffitt Cancer Center and Research Institute and the University of Florida Health Cancer Center involving diabetic patients with advanced-stage (III or IV) prostate cancer. All patients were on hypoglycemic therapy and received either surgery, radiation therapy, or both for their prostate cancer. The control group included patients on metformin, and the study group included patients on a DPP4 inhibitor. Patient data were analyzed from the time of diagnosis until the end of the follow-up period in February 2020. The study protocol was approved by the respective Institutional Review Boards at each institution.

Inclusion criteria included patients with pathologically confirmed prostate cancer and type two diabetes who were started on either metformin or a DPP4 inhibitor at any point during our 19-year observational period from February 2001 to February 2020. Exclusion criteria included patients who were started on metformin or DPP4 inhibitor after progression of their prostate cancer, or those who progressed within less than one month of hypoglycemic agent initiation.

Baseline characteristics were collected at the time of patient enrollment including age, race, and stage of prostate cancer at diagnosis. Data regarding surgical intervention (prostatectomy) or delivery of radiation therapy were also collected. PSA values at diagnosis, after prostatectomy and/or radiation therapy, and at time of biochemical recurrence were collected to determine progression. Progression, or biochemical recurrence, was defined as a rise in PSA of 0.2 ng/mL or greater above nadir after radical prostatectomy, or a rise of 2 ng/mL or greater after radiation therapy.

A total of 161 patients met eligibility criteria and were included in the final analysis, including 120 (75%) patients on metformin and 41 (25%) patients on a DPP4 inhibitor. Roughly half of patients (n = 79, 49%) received radiation therapy during follow-up. The median time of follow-up until progression was 2.2 years (range: 0.1-13.2 years). PFS was calculated for both groups, and Cox proportional hazards regression was used to determine statistical significance. Kaplan-Meier survival analysis was done to predict the effect of DPP4 inhibition and radiation therapy on PFS.

Statistical analysis

For descriptive analysis, continuous variables were presented as mean ± standard deviation, while categorical variables were presented as count (percentage). Mean values of continuous variables were compared using the independent t-test. Categorical variables were compared using the Chi-square test. Cox proportional hazards regression was used to analyze the predicted effects of different variables on the progression of prostate cancer by generating hazard ratios (HRs). A p-value of less than 0.05 was used to indicate statistical significance. A multivariable Cox model was created using all variables reaching statistical significance on univariate analysis. Kaplan-Meier survival analysis was done to predict the effect of DPP4 inhibitor and radiation therapy on PFS. Given the significance of radiation therapy, further subgroup analysis was done using stratified Cox models and Kaplan-Meier analysis. All statistical analysis was performed using SPSS software, version 26 (IBM Corp., Armonk, NY, USA).

## Results

Our study population consisted of 161 patients, including 120 (75%) on metformin and 41 (25%) on a DPP4 inhibitor. About half of the patients (n = 79, 49%) received radiation therapy. Table 1 shows the baseline characteristics of patients grouped by hypoglycemic agent and use of radiation therapy.

**Table 1:** Descriptive analysis of study population, grouped into those on DPP4 inhibitor versus metformin, and those who received radiation versus those who did not. Data presented as mean ± standard deviation for continuous variables, or count (percentage) for categorical variables. P-values are based on Chi-square test or independent t-test DPP4 = dipeptidyl peptidase-4; NA = not applicable; UF = University of Florida

	All patients (n = 161)	DPP4 inhibitor use			Use of radiation		
		Metformin (n = 120)	DPP4 inhibitor (n = 41)	P-Value	Radiation (n = 79)	No radiation (n = 82)	P-Value
Age, years	69 ± 9	68 ± 9	71 ± 11	0.085	68 ± 9	69 ± 10	0.538
Race, white	115 (71%)	88 (73%)	27 (66%)	0.360	55 (70%)	60 (73%)	0.618
Stage III	96 (60%)	77 (64%)	19 (46%)	0.045	53 (67%)	43 (52%)	0.058
Stage IV	65 (40%)	43 (36%)	22 (54%)		26 (33%)	39 (48%)	
Use of surgery	87 (54%)	69 (58%)	18 (44%)	0.131	36 (46%)	51 (62%)	0.034
Use of radiation	79 (49%)	63 (53%)	16 (39%)	0.136	NA	NA	NA
DPP4 inhibitor use	41 (25%)	NA	NA	NA	16 (20%)	25 (30%)	0.136
UF Shands	96 (60%)	66 (55%)	23 (56%)	0.903	54 (68%)	35 (43%)	0.001
Moffitt	65 (40%)	54 (45%)	18 (44%)		25 (32%)	47 (57%)	

**Figure 1:**
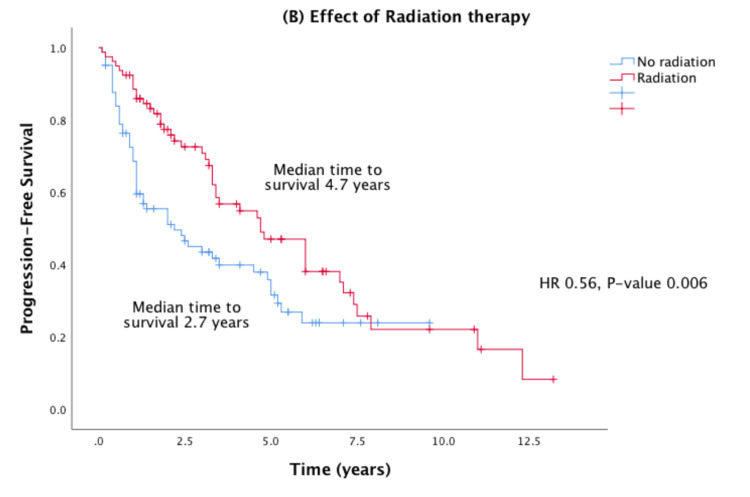
Effects of radiation therapy on Kaplan-Meier PFS in advanced prostate cancer. HR = hazard ratio; PFS = progression-free survival

No significant difference in progression of prostate cancer was found between those on DPP4 inhibitor versus metformin. Median time to progression (TTP) was 3.3 years in the DPP4 group versus 3.5 years in the metformin group. Radiation therapy was associated with improved PFS (HR: 0.56; 95% confidence interval [CI]: 0.37-0.84; p = 0.0006) (Figure 1).

**Figure 2:**
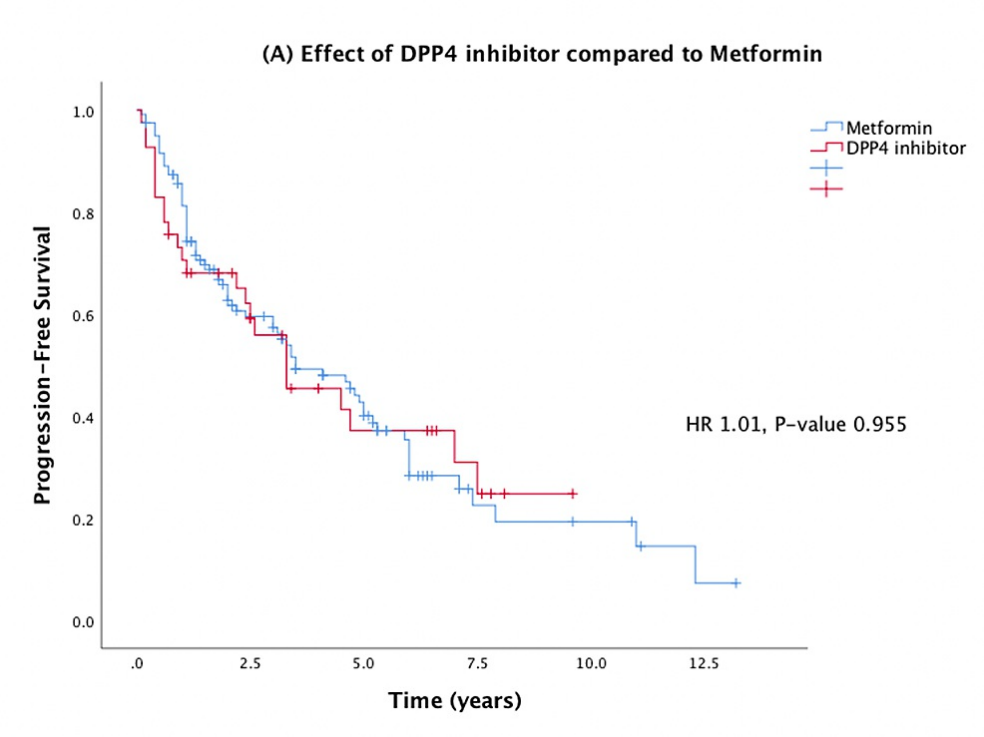
Effects of DPP4 inhibitor compared to metformin on Kaplan-Meier PFS in prostate cancer. HR = hazard ratio; DPP4 = dipeptidyl peptidase-4; PFS = progression-free survival

On Cox proportional hazards regression analysis, there was no significant difference between the use of a DPP4 inhibitor and metformin on the progression of prostate cancer. Specifically, 24 of 41 (59%) patients on DPP4 inhibitors progressed, while 73 of 120 (61%) patients on metformin had progression of prostate cancer (HR: 1.01; 95% CI: 0.64-1.61; p = 0.955) (Figure 2). However, the use of radiation therapy was associated with less progression of disease (HR: 0.56; 95% CI: 0.37-0.84; p = 0.006). Interestingly, young age (defined as age ≤65 years) was associated with worse progression of disease (HR: 1.88; p = 0.002). Stage IV disease (compared to stage III) was associated with worse PFS (HR: 2.59; p < 0.001). Univariate and multivariate analyses are shown in Table 2.

**Table 2:** Analysis of the predicted effect of different variables on progression of prostate cancer using Cox proportional hazards regression on univariate and multivariable analysis. Data presented as mean ± standard deviation for continuous variables, or count (percentage) for categorical variables. P-values are based on Chi-square test or independent t-test CI = confidence interval; DDP4 = dipeptidyl peptidase-4

Variable	Progression of prostate cancer					
	Univariate analysis			Multivariable analysis		
	Hazard ratio	95% CI	P-Value	Hazard ratio	95% CI	P-Value
Age ≤65 years	1.8	1.21-2.68	0.004	1.88	1.26-2.80	0.002
White race	0.8	0.52-1.22	0.298			
Stage IV cancer	2.49	1.66-3.72	<0.001	2.59	1.73-3.89	<0.001
Use of surgery	0.69	0.46-1.03	0.068			
Use of radiation	0.59	0.39-0.89	0.012	0.56	0.37-0.84	0.006
DPP4 inhibitor use	1.01	0.64-1.61	0.955			

Median PFS for the entire prostate cancer cohort was 3.5 years (2.4-4.6 years), 3.3 years in the DPP4 group versus 3.5 years in the metformin group. The use of a DPP4 inhibitor did not significantly impact PFS compared to the use of metformin. The use of radiation therapy showed a significant improvement in PFS (HR: 0.56; 95% CI: 0.37-0.84; p = 0.0006). These PFS outcomes are demonstrated in Figure 2.

**Figure 3:**
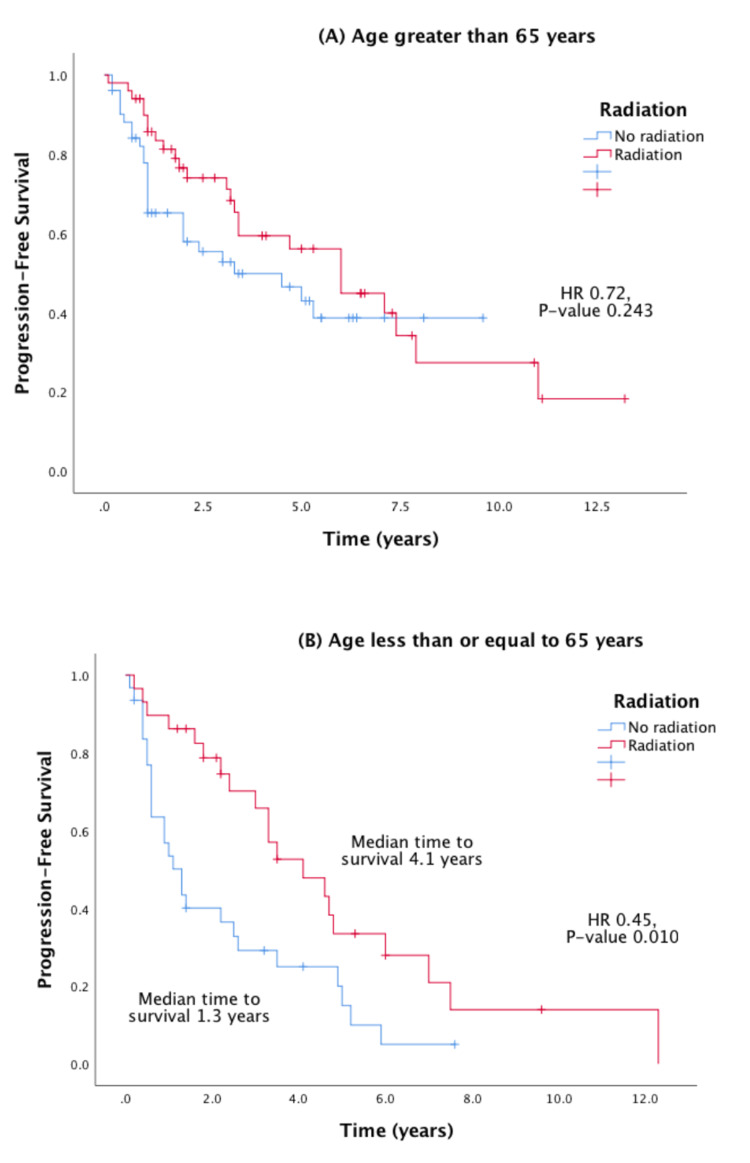
Effect of radiation therapy on Kaplan-Meier PFS in prostate cancer stratified by age (panels A and B). HR = hazard ratio; PFS = progression-free survival

**Figure 4:**
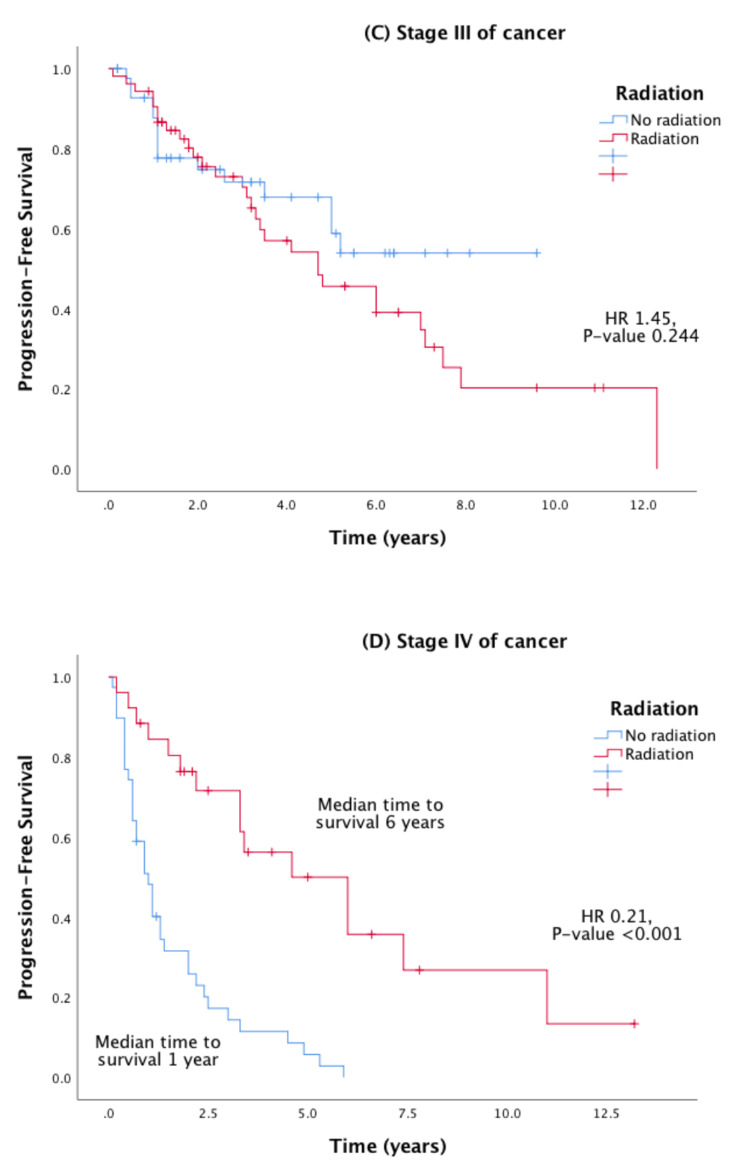
Effect of radiation therapy on Kaplan-Meier PFS in prostate cancer stratified by stage of cancer (panels C and D). HR = hazard ratio; PFS = progression-free survival

Stratified Cox regression and Kaplan-Meier analysis were performed to evaluate the PFS effect of radiation therapy stratified by age, stage of cancer, and medical center. These stratified survival curves are shown in Figures 3, 4, and show a favorable PFS when radiation therapy was used in younger patients and patients with stage IV cancer.

## Discussion

Though the exact mechanism is unknown, there have been numerous proposed mechanisms for the role of DPP4 in cancer. CD26/DPP4 carries immunological functions, including T-cell activation and signal transduction, thus upregulating downstream inflammatory chemokines such as TNF-α and IFN-γ. CD26/DPP4 also has a well-established role in cancer metastasis [2,7,8]. CD26/DPP4 stimulates the activation of matrix metalloproteinase-9 (MMP9), which degrades the protective extracellular matrix that allows cells to invade. Therefore, inhibiting CD26/DPP4 has been proposed to delay cancer cell invasion or metastasis [5]. Furthermore, CD26/DPP4 can bind extracellular matrix proteins such as collagen and fibronectin, which facilitate tumor invasion. In addition, CD26/DPP4 cleaves ligands CXCL10 and CXCL12, which play a role in lymphocyte migration and tumor immune surveillance, thus increasing susceptibility to metastasis [3].

CD26/DPP4 expression was identified in numerous tumors, such as MPM, RCC, GIST, HCC, and colorectal, lung, prostate, and ovarian cancer. The first in human phase one clinical trial of humanized anti-CD26 monoclonal antibody (YS110) found prolonged disease stabilization in patients with advanced or refractory mesothelioma [2].

Bishnoi et al. demonstrated that diabetic patients with colorectal cancer or lung cancer who were treated with DPP4 inhibitors had a statistically significant survival advantage (HR: 0.89; 95% CI: 0.82-0.97; p = 0.0007). Patients on both DPP4 inhibitors and metformin, which has also been associated with tumor suppression, had even more pronounced of a survival advantage, suggesting a synergistic effect (HR: 0.83; 95% CI: 0.77-0.90; p < 0.0001) [4]. In a multi-center retrospective study of diabetic patients with advanced colorectal or airway (lung, head, and neck) cancer, only 23.7% of patients on DPP4 inhibitor and metformin exhibited cancer progression compared to 50.9% of controls on metformin and a sulfonylurea (odds ratio: 0.30; 95% CI: 0.11-0.81; p = 0.010) [3].

The antineoplastic effects of metformin has been previously established in numerous cancers, including prostate cancer [5,9,10]. Through the adenosine monophosphate-activated protein kinase (AMPK)-dependent suppression of androgen signaling pathway and insulin-like growth factor-1 (IGF-1), metformin inhibits proliferation of prostate cancer. In addition, metformin improves immune response by increasing CD8+ tumor-infiltrating lymphocytes and decreasing inflammatory chemokine production of IL-2, TNFα, and IFNγ [9,10].

Using the SEER-Medicare database, Shah et al. demonstrated that prostate cancer patients on DPP4 inhibitors alone had improved survival compared to those who were not on DPP4 inhibitors, independent of metformin (HR: 0.77; 95% CI: 0.64-0.93; p = 0.0005). It was proposed that this advantage may be partially explained by the expression pattern of CD26/DPP4 in malignant prostate cells [5]. A study showed that prostate cancer tissue with high DPP4 expression was associated with poor prognosis (p < 0.0001) [6]. Metformin alone also exhibited a significant OS benefit in patients with prostate cancer with HR of 0.87 (95% CI: 0.81-0.93; p < 0.0001) [5].

This study aims to further investigate the clinical impact of using DPP4 inhibitors in advanced prostate cancer using clinical data available at two tertiary referral cancer institutions. It is also the first study to our knowledge that directly compares the effects of metformin with DPP4 inhibitors in prostate cancer. While prior studies have suggested possibly synergistic survival benefits of metformin and DPP4 inhibitors in cancer, data directly comparing the two drugs have been limited [4,5]. In our multi-center retrospective cohort analysis, DPP4 inhibitor use compared to metformin did not show a significant PFS benefit in advanced-stage prostate cancer. One potential explanation for the non-statistically significant results may be attributed to the relatively small sample size and the retrospective design of the study which lends itself to patient selection bias. We also found that radiation therapy improves PFS in prostate cancer, with more significant impact in younger patients and patients with stage IV cancer (Figures 2, 3).

Our analysis has various limitations, most notably the sample size. Because DPP4 inhibitors are typically used as second or third-line agents for glycemic control in type two diabetes, a small sample size of diabetic patients on DPP4 inhibitors who had advanced-stage prostate were identified. Our relatively small sample size may explain our statistically non-significant outcomes in PFS. This also led to a sample size discrepancy between the two treatment groups, with more patients in the metformin group compared to DPP4 inhibitor group.

The interplay between the effects of DPP4 inhibitors on the androgen receptor (AR) is also important to note. It has been established that the initial treatment for metastatic prostate cancer is through androgen deprivation (ADT). Preclinical models have shown that AR activity was persistent in cases with decreased DPP4 function, thereby functioning as an AR-regulated suppressor gene [11]. This suggests that inhibition of DPP4 with this targeted class of anti-diabetic drugs may provide a resistance mechanism in patients with metastatic prostate cancer.

While the exact mechanism of interplay between the AR and DPP4 inhibitors is still in question, it is important to note that the Russo et al. study, which suggested a potential resistance mechanism, is the only such study to date to posit such a hypothesis, while the Shah et al. SEER-Medicare study found the opposite, reporting that patients with prostate cancer on DPP4 inhibitors had superior survival outcomes [5]. As our study showed equivocal findings, this is very important data, as unlike other tumor types (colorectal, lung, head, and neck) which showed survival benefit with DPP4 inhibitors both in single and multi-center retrospective analyses as well as on large-scale SEER-Medicare database queries, there is a discrepancy in the findings regarding the impact on survival of DPP4 inhibitors in prostate cancer. The impact of the molecular interplay between the AR and DPP4 is of utmost importance to clarify and further understand the impact of these commonly used diabetic drugs on the most frequent cancer in males [12]. Further large-scale efforts are necessary to investigate the survival outcomes in patients with advanced prostate cancer on metformin versus DPP4 inhibitors. It will also be important to determine if this is applicable to prostate cancer as a whole, or if this effect is specific to castrate-sensitive prostate cancer versus castrate-resistant prostate cancer.

## Conclusions

Contrary to what has been shown in patients with diabetes and advanced colorectal or airway cancers, exposure to DPP4 inhibitors did not lead to statistically significant improvement in PFS in patients with advanced-stage prostate cancer. Recent data suggest that DPP4 may act as a tumor suppressor gene to the AR pathway, and that DPP4 inhibition can result in emergence of resistance to ADT. Further investigation regarding this molecular interplay is necessary to determine the effects on survival on this prevalent malignancy.
